# Association of anemia with mortality in young adult patients with intracerebral hemorrhage

**DOI:** 10.1038/s41598-023-46941-z

**Published:** 2023-11-12

**Authors:** Yixin Tian, Yu Zhang, Jialing He, Pengfei Hao, Tiangui Li, Yangchun Xiao, Liyuan Peng, Yuning Feng, Xin Cheng, Haidong Deng, Peng Wang, Weelic Chong, Yang Hai, Lvlin Chen, Chao You, Fang Fang

**Affiliations:** 1 West China Hospital, Sichuan University, No. 37, Guo Xue Xiang, Chengdu, 610041 Sichuan China; 2 Affiliated Hospital of Chengdu University, Chengdu, Sichuan China; 3 The Second Affiliated Hospital of Guangzhou Medical University, Guangzhou, Guangdong China; 4 Shanxi Provincial People’s Hospital, Taiyuan, Shanxi China; 5 The First People’s Hospital of Longquanyi District, Chengdu, Sichuan China; 6 Thomas Jefferson University, Philadelphia, PA USA

**Keywords:** Neurological disorders, Stroke

## Abstract

This study aimed to examine the association of hemoglobin concentration with a 90-day mortality of young adult patients with ICH in a large retrospective cohort. A retrospective observational study was conducted between December 2013 and June 2019 in two tertiary academic medical centers in China. We defined patients with hemoglobin concentration < 80 g/L as severe anemia and 80–120/130 g/L as mild to moderate anemia. We also defined patients with hemoglobin concentration > 160 g/L as high hemoglobin. Associations of hemoglobin and outcomes were evaluated in multivariable regression analyses. The primary outcome was mortality at 90 days. We identified 4098 patients with ICH who met the inclusion criteria. After adjusting primary confounding variables, the 90-day mortality rate was significantly higher in young patients with severe anemia (OR, 39.65; 95% CI 15.42–101.97), moderate anemia (OR, 2.49; 95% CI 1.24–5.00), mild anemia (OR, 1.89; 95% CI 1.20–2.98), and high hemoglobin (OR, 2.03; 95% CI 1.26–3.26) group than in young patients of the normal group. The younger age was associated with a higher risk of death from anemia in patients with ICH (P for interaction = 0.01). In young adult patients with ICH, hemoglobin concentration was associated with 90-day mortality, and even mild to moderate anemia correlated with higher mortality. We also found that in ICH patients with anemia, younger age was associated with higher risk.

## Introduction

Intracerebral hemorrhage (ICH) is a common cause of stroke (15–30%), which affects about 2.5 per 10,000 people each year, and only 20% of survivors are functionally independent at 6 months^1,2^. ICH patients with comorbidities, such as severe anemia, are likely to have a poor prognosis and be admitted to an intensive care unit^3,4^.

Although evidence has demonstrated that anemia is associated with poor outcomes in patients with ICH^5^, however, age differences in the association need to be more fully characterized. ICH and anemia are more common in the elderly, but there is still a large amount of young adult ICH patients with anemia (from 12.9 to 31%)^6–8^. The capacity of younger adults to increase cardiac output is considerably higher than that of older adults^9^, and regulation of cerebral oxygenation alters with increasing age^10^. This evidence suggests that youth have more tolerance for a reduction in Hb. Moreover, several guidelines recommend that transfusion be not only based on the hemoglobin concentration but also on consideration of overall clinical status, of which age is one of the important factors^11^. Advancements in our understanding of the relationship between age, anemia, and outcomes in patients with ICH may potentially inform the successful development of blood transfusion services.

The objectives of this study were to examine the association of hemoglobin concentration with a 90-day mortality of young adult patients with ICH in a large retrospective cohort. We hypothesized both low and high hemoglobin concentrations on admission were associated with high mortality in young adult patients with ICH.

## Methods

### Study design and setting

This is a retrospective, observational study based on consecutively electronic health records from West China Hospital Sichuan University between December 2010 and July 2019 and the First of People’s Hospital of Longquanyi District between January 2017 and October 2020. This study was approved by both West China Hospital and the First of People’s Hospital of Longquanyi District Institutional Review Board. All authors had no access to information that could identify individual participants during or after data collection.

### Participants

Patients through the following were included for further analysis: (1) radiologically confirmed case of acute primary (non-traumatic) ICH, (2) no history of previous ICH, (3) follow-up data available at 90 days.

Patients were excluded if they met the critical exclusion criteria: (1) traumatic ICH, (2) acute ischemic stroke, (3) subarachnoid hemorrhage, (4) subdural hemorrhage, (5) first computed tomography (CT) digital imaging and communication not available in electronic medical records, (6) unavailable hemoglobin concentration at admission.

Patients younger than 50 were defined as young adult patients, and older than 50 were defined as elderly patients.

### Hemoglobin on admission

We used hemoglobin on admission as the exposure factor in this study. As per the guideline of WHO^12^, the diagnosis of anemia in men was based on a hemoglobin of less than 130 g/L; in women, it was less than 120 g/L. Multiple guidelines recommend the hemoglobin threshold for red blood cell transfusion is 70–80 g/L for adults. Thus, we defined patients with hemoglobin concentration < 80 g/L as severe anemia and 80–120/130 g/L as mild to moderate anemia. We also defined patients with hemoglobin concentration > 160 g/L as high hemoglobin.

### Outcomes

The primary outcome was mortality at 90 days. Secondary outcomes were mortality at 30 days, 1 year, and the longest follow-up.

We retrieved the whole death records up to August 15, 2021, for further analysis via the Household Registration Administration System from the Department of Public Security, Sichuan Province, China. China’s law demands that once a citizen dies, the head of household, relatives, dependents, or neighbors shall report the death registration to the household registration authority and cancel the household registration within 1 month. This system has accurate death records. Therefore, it was negligible for the rate of loss to follow up this study.

### Statistical analysis

Continuous variables were presented as mean ± standard deviation (SD), and categorical variables were reported with frequencies and percentages to describe the baseline characteristics of enrolled patients. The missing values have been replaced by mean values.

Univariable logistic regression analysis was performed to assess the association of baseline characteristics with favorable outcomes. Confounders included in the multivariable regression models were derived from previous relative studies and clinical expertise, including age, gender, smoking, alcohol abuse, hypertension, diabetes, size of the hematoma, infratentorial hematoma, intraventricular hematoma, Glasgow Coma Scale (GCS). We used restricted cubic spline functions to analyze the log-linearity assumption for continuous characteristics. Then, factors associated with a value of P < 0.10 in univariate analyses were implemented into the multivariable logistic regression model. We also performed subgroup comparisons between patients with low hemoglobin concentration and normal hemoglobin concentration at admission by age stratification.

Kaplan–Meier analyses and log-rank tests were used to examine all-cause mortality after ICH amongst different subgroups of hemoglobin concentration. Cox’s proportional hazards model was used to adjust for major potential confounders and estimate hazard ratios (HR) for survival.

P values less than 0.05 were set as the threshold for statistical significance, and all P values were 2-sided. R statistical software (version 4.1.3; Foundation for Statistical Computing) was used to perform all statistical analyses.

### Ethical statement

This is a retrospective study based on medical records and has been granted an exemption from requiring written informed consent by the ethics committee of West China Hospital (No. 20211701) and the ethics committee of the First of People’s Hospital of Longquanyi District (No. 2022004). This study complied with the guidelines for human studies and was conducted ethically in accordance with the World Medical Association Declaration of Helsinki.

## Results

Of the 7013 patients diagnosed with ICH in the above two medical hospitals. After excluding criteria, a total of 1290 young adult patients (the age from 18 to 50 years) and 2808 elderly patients (> 50 years) were included in this study. A flowchart of patient selection is shown in eFigure 1. The demographic characteristics of recruited patients are shown in Table 1 (the detailed characteristics of young adult and elderly patients were shown in eTable 1 and eTable 2). Among the 1290 young adult patients, 360 (28%) had anemia on admission (Hb concentration: < 120 for females, < 130 for males). Patients with lower hemoglobin concentration were more likely to be female and presented with fewer smokers and alcohol abusers, more hypertension, larger hematoma, and a decline in GCS (P < 0.05).

**Table 1:** Baseline characteristics of the patients stratified by baseline hemoglobin.

Characteristics	Hemoglobin (g/L)					
	Severe anemia* N = 98	Moderate anemia* N = 220	Mild anemia* N = 1073	Normal* N = 2321	High hemoglobin* N = 386	P
Age, year, mean (SD)	54.70 (16.07)	58.30 (17.59)	61.72 (15.11)	57.78 (14.31)	51.48 (12.78)	< 0.001
Female, n (%)	50 (51.0)	112 (50.9)	570 (53.1)	609 (26.2)	21 (5.4)	< 0.001
Smoking, n (%)						< 0.001
Never	79 (80.6)	175 (79.5)	841 (78.4)	1507 (64.9)	195 (50.5)	
Current	15 (15.3)	36 (16.4)	189 (17.6)	680 (29.3)	167 (43.3)	
Ever	4 4.1)	9 (4.1)	43 (4.0)	136 (5.9)	24 (6.2)	
Alcohol abuse, n (%)	22 (22.4)	35 (15.9)	209 (19.5)	779 (33.5)	197 (51.0)	< 0.001
Medical history, n (%)						
Hypertension	60 (61.2)	142 (64.5)	683 (63.7)	1714 (73.8)	290 (75.1)	< 0.001
Diabetes	15 (15.3)	36 (16.4)	116 (10.8)	213 (9.2)	23 (6.0)	< 0.001
Hematoma characteristics						
Size of hematoma, ml, mean (SD)	23.12 (21.81)	36.41 (36.07)	24.70 (29.54)	21.78 (28.37)	26.23 (29.45)	< 0.001
Infratentorial location, n (%)	9 (9.2)	35 (15.9)	186 (17.3)	449 (19.3)	92 (23.8)	0.004
Intraventricular location, n (%)	29 (29.6)	59 (26.8)	263 (24.5)	548 (23.6)	106 (27.5)	0.3
Glasgow Coma Scale, mean (SD)	8.63 (4.66)	8.98 (4.36)	10.76 (4.17)	11.28 (3.97)	10.58 (4.45)	< 0.001

*Severe anemia: < 80; Moderate anemia: 81–100. Mild anemia: 101–120 for females, 101–130 for males.

*Normal: 121–160 for females, 131–160 for males; High hemoglobin: > 160.

Univariate and multivariable analysis results are summarized in Table 2 to demonstrate the associations between hemoglobin of admission and mortality at 90 days. After adjusting for confounders (age, smoking, hypertension, size of hematoma, intraventricular hematoma, GCS, operation of hematoma), the 90-day mortality rate was significantly higher in young adult patients with severe anemia, mild to moderate anemia, and high hemoglobin group than the normal group (Severe anemia versus normal: adjusted odds ratio (aOR), 39.65; 95% CI 15.42–101.97; moderate anemia versus normal: aOR, 2.49; 95% CI 1.24–5.00, mild anemia versus normal: aOR, 1.89; 95% CI 1.20–2.98, high hemoglobin versus normal: aOR, 2.03; 95% CI 1.26–3.26). We found the association between hemoglobin concentration and 90-day mortality were more obvious in young adult patients than elderly (The aOR in young patients: 39.65 (15.42–101.97) versus the aOR in elder patients: 3.35 (1.79–6.30)). As shown in Fig. 2, with the graded decrease in hemoglobin concentrations, the risk of death gradually increased.

**Table 2:** Unadjusted and adjusted associations between admission hemoglobin and mortality at 90 days based on different cutoff values.

Characteristics		Hemoglobin (g/L)	Events, n (%)	Unadjusted OR	Multivariable regression adjusted OR	P
Young adult patients	Continuous	Per SD	NA	0.69 (0.61–0.79)	0.71 (0.61–0.84)	< 0.001
	Quartile	Normal^#^	101/731 (13.8%)	1 (Reference)	1 (Reference)	< 0.001
		Severe anemia	32/41 (78%)	19.71 (9.16–42.43)	39.65 (15.42–101.97)	
		Moderate anemia	23/69 (33.3%)	2.77 (1.62–4.75)	2.49 (1.24–5.00)	
		Mild anemia	62/250 (24.8%)	1.83 (1.29–2.60)	1.89 (1.20–2.98)	
		High hemoglobin	57/199 (28.6%)	2.23 (1.54–3.21)	2.03 (1.26–3.26)	
Elderly patients	Continuous	Per SD	NA	0.66 (0.58–0.74)	0.71 (0.62–0.82)	< 0.001
	Quartile	Normal^#^	311/1553 (20%)	1 (Reference)	1 (Reference)	< 0.001
		Severe anemia	30/57 (52.6%)	4.13 (2.42–7.04)	3.35 (1.79–6.30)	
		Moderate anemia	76/151 (50.3%)	3.77 (2.68–5.30)	2.42 (1.62–3.63)	
		Mild anemia	207/823 (25.2%)	1.25 (1.02–1.52)	1.13 (0.89–1.43)	
		High hemoglobin	45/187 (24.1%)	1.18 (0.83–1.68)	0.92 (0.60–1.42)	

*Severe anemia: < 80; Moderate anemia: 81–100. Mild anemia: 101–120 for females, 101–130 for males.

*Normal: 121–160 for females, 131–160 for males; High hemoglobin: > 160.

^#^The control group was a normal hemoglobin concentration group (121–160 for females, 131–160 for males).

**Figure 1:**
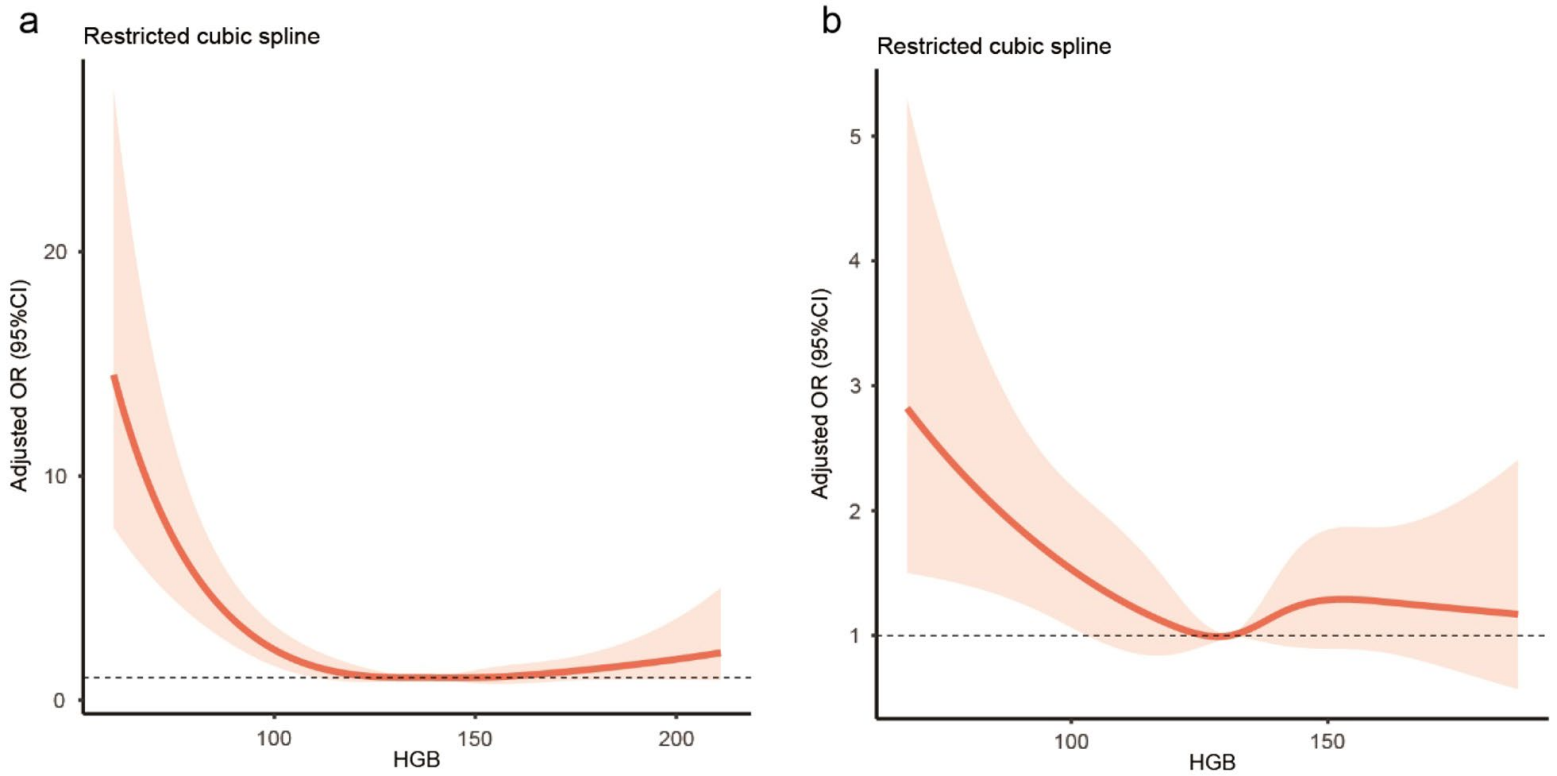
Relationship between hemoglobin and 90-day mortality in patients with ICH. (**a**) Young adult patients, (**b**) elderly patients.

The restricted cubic spline was used to visualize the association of hemoglobin concentration with 90-day mortality. A nonlinear association between Hb and 90 days mortality was presented in Fig. 1 (P for non-linearity < 0.001)^13,14^. We set a Hb cutoff of 140 g/L in the young adult patient group and found both below and above the cutoff showed a strong and graded association between Hb concentration and mortality (below the cutoff: adjusted OR 0.47, 95% CI 0.38–0.59, for each 1-SD Hb increase; above the cutoff: adjusted OR 1.72, 95% CI 1.36–2.17, for each 1-SD Hb increase). When hemoglobin was analyzed as continuous, the adjusted OR of 90-day mortality for each 1-SD increase in hemoglobin was 0.71 (95% CI 0.61–0.84).

**Figure 2:**
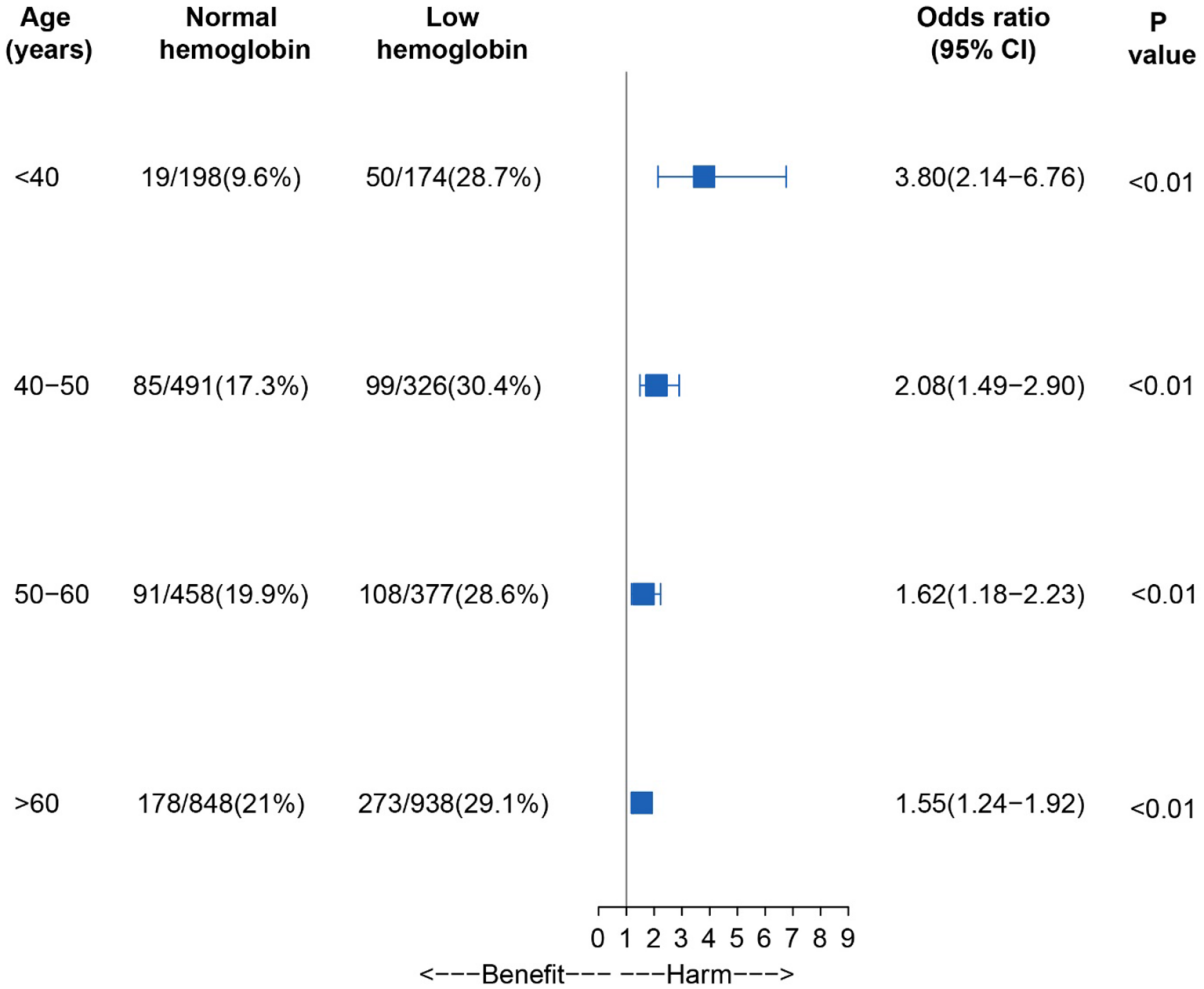
Subgroup analysis of the association between hemoglobin concentration and 90-day mortality in age stratification.

**Figure 3:**
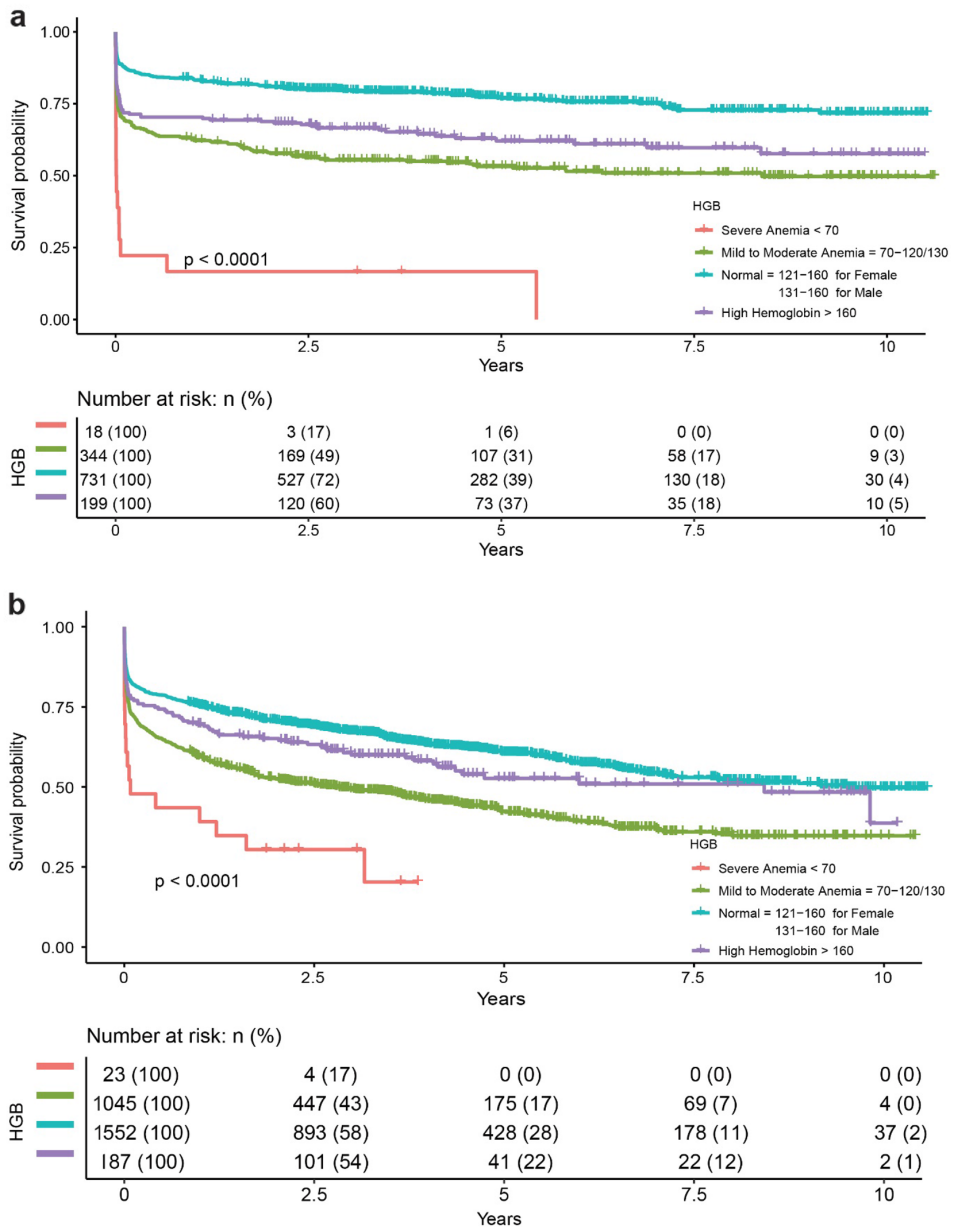
Kaplan–Meier survival curves indicating the relationship between admission hemoglobin and 90-day mortality after ICH. (**a**) Young adult patients, (**b**) elderly patients.

We conducted subgroup analysis by age stratification on hemoglobin concentration with a cutoff value of 130 g/L, and we found that the mortality was higher in young ICH patients with anemia (Fig. 2, P for interaction = 0.01). The Kaplan–Meier curves indicated that either lower or higher hemoglobin concentration of admission was associated with higher mortality after ICH (P < 0.0001; Fig. 3). Furthermore, we collected serial hemoglobin measurements of patients in ICH after admission. Similar results of mortality at 30 days, 1 year, and the longest follow-up were shown in eTable 3 in the appendix. During hospital admission within the first week, the hemoglobin concentration of those who died was generally lower than those who survived (eFigure 2; P < 0.001).

## Discussion

In this cohort study, we found both low and high hemoglobin concentrations on admission indicated poor outcomes in young adult patients with ICH. The survival analysis showed even mild to moderate anemia correlated with higher mortality. Subgroup analysis showed that the effect of anemia on mortality increases with decreasing age.

Extensive researchers have reported the association of anemia with the mortality of ICH patients. Miguel et al. demonstrated the association of anemia on admission with greater intracerebral hemorrhage severity, higher mortality, and worse functional outcomes^11^. Meanwhile, Tiffany et al. suggested that nadir hemoglobin could also be used to predict poor functional outcomes of ICH patients^15–17^. All the above studies have specifically emphasized the importance of anemia in the outcome of patients with ICH.

The reason for anemia-related mortality in ICH patients is complex, and age is one of the most important factors^16,18^. Previous studies indicated the etiologies and risk factors of anemia at younger ages are different from those in older people. Compared with elderly patients, young adult patients were more likely with clonal disorders of hematopoiesis, yet the vast majority of elderly patients with nutritional deficiency anemias resulting from chronic systemic syndromes such as renal insufficiency^19,20^. The damage of anemia in young adult patients was more severe than in other age stratification. Therefore, the results of cohort studies in elderly patients cannot directly be translated to apply to younger individuals.

However, none of these studies focused on young adult ICH patient groups. Multiple large retrospective studies indicated the etiology, diagnosis, and prognosis of anemia in young adults and the elderly are different^21^. Besides these differences, choosing young adult patients as the study sample could reduce the confounding effects of age, such as mild anemia caused by the age-associated decline in renal function or in testosterone concentrations^22^. To our knowledge, this research was the first large cohort study to conduct the association of anemia with the prognosis in young adult ICH patients. We found both low and high hemoglobin concentration on admission associated with poor outcomes in young adult ICH patients, and even mild to moderate anemia correlated with higher mortality. Within the first week after admission, the trajectory of hemoglobin concentration showed a continuous downward trend, which indicates the necessity of early intervention of anemia among young adult patients with ICH. As shown in Fig. 3, risk of death seems to stabilize in younger patients early whereas it continues to decline in older patients as time passes. That may be due to deaths from other causes in older individuals such as advanced cancers.

Our study represents the largest study of young adults to date. A total of 1290 young adults and 2808 elderly ICH patients with anemia were included in cohorts. Each sample contains detailed medical records and accurate death records, which are extracted from household registration data. There are some aspects of the implications in this study. First, including younger patients allows for a more comprehensive understanding of the disease’s impact across different age groups. By comparing outcomes between younger and older patients, researchers can more accurately gauge the disease’s progression and its potential influence on mortality rates. Second, although the causes of anemia and causes of death cannot be provided, it is still valuable to collect data on younger patients. This data could help clinicians better understand the prognosis and potential outcomes for younger patients with ICH. By building a more comprehensive dataset across age groups, healthcare providers can make more informed decisions regarding treatment options, patient monitoring, and prognosis discussions. While it is true that causes of anemia and causes of death cannot be provided, the inclusion of younger patients can still contribute to a more thorough understanding of mortality following ICH.

There are also some limitations in this study. First, although we adjusted for confounding factors by multivariable logistic regression, there might be several unobserved confounders, such as etiology, time of admission, and surgical intervention. Second, the outcome of this study did not contain functional outcomes such as the modified Rankin Scale. Thirdly, we did not distinguish between acute and chronic anemia in patients with ICH. Fourth, due to the inherent limitations of retrospective clinic studies, the causes of anemia, causes of death, rates of blood transfusion, and other severe comorbidities (advanced cancer) are not identifiable in the data presented.

## Conclusions

In this large study, we collected 1290 young adults and 2808 elderly patients with ICH and found that hemoglobin concentration on admission was associated with 90-day mortality. Those results indicated both low and high hemoglobin concentrations on admission presented negative effects and even mild to moderate anemia correlated with higher mortality in young adult patients with ICH. Compared to older age, we found that the mortality was higher in young ICH patients with anemia. Further studies may be conducted on early intervention of anemia in young adult patients with ICH.

### Supplementary Information

Supplementary Information.

## Data Availability

The datasets used and/or analyzed during the current study are available from the corresponding author upon reasonable request.
