# Supplementary material for: Association of anemia with mortality in young adult patients with intracerebral hemorrhage
Source: Sci Rep. 2023 Nov 12;13:19711. doi: 10.1038/s41598-023-46941-z (PMC10641070; doi:10.1038/s41598-023-46941-z)
Supplement: Supplementary file 1 — Supplementary Information. [file 41598_2023_46941_MOESM1_ESM.docx]

**Append**

**eTable 1 Baseline characteristics of the patients stratified by baseline hemoglobin in young adult patients**

**eTable 2 Baseline characteristics of the patients stratified by baseline hemoglobin in elderly patients**

**eTable 3 Associations Between Continuous and Quintile of admission hemoglobin and mortality**

**eFigure 1 Flow chart of enrollment**

**eFigure 2 Trajectory of hemoglobin concentration within the first week after admission among young adult and elderly patients with ICH.**

**eTable 1 Baseline characteristics of the patients stratified by baseline hemoglobin in young adult patients.**

| Characteristics |  | | Hemoglobin (g/L) | | | | |
| --- | --- | --- | --- | --- | --- | --- | --- |
|  | Severe Anemia* N=41 | Moderate anemia* N=69 | | Mild  anemia* N=250 | Normal*  N=731 | High hemoglobin* N=199 | P |
| Age, year, mean (SD) | 39.85 (9.03) | 37.94 (9.40) | | 40.98 (8.05) | 41.30 (7.43) | 41.51 (6.54) | <0.001 |
| Female, n (%) | 21 (51.2) | 40 (58.0) | | 132 (52.8) | 151 (20.6) | 6 ( 3.0) | <0.001 |
| Smoking, n (%) |  |  | |  |  |  | <0.001 |
| Never | 34 (82.9) | 53 (76.8) | | 199 (79.6) | 455 (62.1) | 93 (46.7) |  |
| Current | 5 (12.2) | 16 (23.2) | | 47 (18.8) | 256 (34.9) | 101 (50.8) |  |
| Ever | 2 ( 4.9) | 0 ( 0.0) | | 4 ( 1.6) | 22 ( 3.0) | 5 ( 2.5) |  |
| Alcohol abuse, n (%) | 10 (24.4) | 13 (18.8) | | 48 (19.2) | 278 (37.9) | 102 (51.3) | <0.001 |
| Medical history, n (%) |  | |  | | | | |
| Hypertension | 23 (56.1) | 30 (43.5) | | 136 (54.4) | 465 (63.4) | 141 (70.9) | <0.001 |
| Diabetes | 2 ( 4.9) | 1 ( 1.4) | | 15 ( 6.0) | 30 ( 4.1) | 5 ( 2.5) | 0.301 |
| Hematoma characteristics |  | |  | | | | |
| Size of hematoma, ml,  mean (SD) | 27.01 (23.87) | 31.16 (32.30) | | 22.68 (27.04) | 19.83 (30.90) | 25.15 (25.62) | 0.019 |
| Infratentorial location, n (%) | 3 ( 7.3) | 15 (21.7) | | 53 (21.2) | 177 (24.1) | 50 (25.1) | 0.123 |
| Intraventricular location, n (%) | 9 (22.0) | 16 (23.2) | | 56 (22.4) | 161 (22.0) | 54 (27.1) | 0.655 |
| Glasgow Coma Scale, mean (SD) | 8.54 (4.69) | 9.68 (4.32) | | 10.42 (4.41) | 11.40 (4.06) | 10.53 (4.45) | <0.001 |
|  |  |  | |  |  |  |  |

*Severe anemia: <80; Moderate anemia: 81-100. Mild anemia: 101-120 for females,101-130 for males.

*Normal: 121-160 for females, 131-160 for males; High hemoglobin: >160.

**eTable 2 Baseline characteristics of the patients stratified by baseline hemoglobin in elderly patients.**

| Characteristics |  | | Hemoglobin (g/L) | | | |
| --- | --- | --- | --- | --- | --- | --- |
|  | Severe Anemia* N=57 | Moderate anemia* N=151 | Mild  anemia* N=823 | Normal*  N=1590 | High hemoglobin* N=187 | P |
| Age, year, mean (SD) | 65.39 (10.57) | 67.61 (11.56) | 68.02 (10.37) | 65.38 (9.52) | 62.10 (8.53) | <0.001 |
| Female, n (%) | 29 (50.9) | 72 (47.7) | 438 (53.2) | 458 (28.8) | 15 ( 8.0) | <0.001 |
| Smoking, n (%) |  |  |  |  |  | <0.001 |
| Never | 45 (78.9) | 122 (80.8) | 642 (78.0) | 1052 (66.2) | 102 (54.5) |  |
| Current | 10 (17.5) | 20 (13.2) | 142 (17.3) | 424 (26.7) | 66 (35.3) |  |
| Ever | 2 ( 3.5) | 9 ( 6.0) | 39 ( 4.7) | 114 ( 7.2) | 19 (10.2) |  |
| Alcohol abuse, n (%) | 12 (21.1) | 22 (14.6) | 161 (19.6) | 501 (31.5) | 95 (50.8) | <0.001 |
| Medical history, n (%) |  | |  | | | |
| Hypertension | 13 (22.8) | 35 (23.2) | 547 (66.5) | 1249 (78.6) | 149 (79.7) | <0.001 |
| Diabetes | 20 (35.1) | 43 (28.5) | 101 (12.3) | 183 (11.5) | 18 ( 9.6) | <0.001 |
| Hematoma characteristics |  | |  | | | |
| Size of hematoma, ml,  mean (SD) | 20.71 (20.32) | 38.66 (37.47) | 25.32 (30.26) | 22.67 (27.10) | 27.33 (32.96) | <0.001 |
| Infratentorial location, n (%) | 6 (10.5) | 20 (13.2) | 133 (16.2) | 272 (17.1) | 42 (22.5) | 0.108 |
| Intraventricular location, n (%) | 20 (35.1) | 43 (28.5) | 207 (25.2) | 387 (24.3) | 52 (27.8) | 0.277 |
| Glasgow Coma Scale, mean (SD) | 8.70 (4.68) | 8.66 (4.36) | 10.86 (4.09) | 11.23 (3.93) | 10.63 (4.45) | <0.001 |
|  |  |  |  |  |  |  |

*Severe anemia: <80; Moderate anemia: 81-100. Mild anemia: 101-120 for females,101-130 for males.

*Normal: 121-160 for females, 131-160 for males; High hemoglobin: >160.

**eTable 3 Associations Between Continuous and Quintile of admission hemoglobin and mortality in young adult patients**

| Outcomes | Hemoglobin | Events, n (%) | Unadjusted OR | Multivariable Regression adjusted OR | P |
| --- | --- | --- | --- | --- | --- |
| Mortality at 30 days |  |  |  |  |  |
| Continuous | per SD | NA | 1.12 (1.10-1.13) | 1.06 (1.03-1.08) | <0.001 |
| Quartile | Normal | 84/731(11.5%) | 1 [Reference] | 1 [Reference] | <0.001 |
|  | Severe anemia | 31/41(75.6%) | 20.57(9.77-43.30) | 32.67(13.30-80.24) |  |
|  | Moderate anemia | 19/69(27.5%) | 2.52(1.43- 4.46) | 1.83( 0.92- 3.64) |  |
|  | Mild anemia | 54/250(21.6%) | 1.83(1.26- 2.65) | 1.63( 1.04- 2.55) |  |
|  | High hemoglobin | 54/199(27.1%) | 2.47(1.69- 3.61) | 2.38( 1.49- 3.81) |  |
| Mortality at one year |  |  |  |  |  |
| Continuous | per SD | NA | 1.11 (1.10-1.13) | 1.06 (1.04-1.08) | <0.001 |
| Quartile | Normal | 119/731(16.4%) | 1 [Reference] | 1 [Reference] | <0.001 |
|  | Severe anemia | 32/40(80%) | 17.92(8.08-39.78) | 29.84(11.37-78.29) |  |
|  | Moderate anemia | 28/69(40.6%) | 3.06(1.83- 5.13) | 2.82( 1.45- 5.48) |  |
|  | Mild anemia | 68/247(27.5%) | 1.70(1.22- 2.38) | 1.55( 1.00- 2.39) |  |
|  | High hemoglobin | 59/199(29.6%) | 1.89(1.32- 2.70) | 1.71( 1.08- 2.72) |  |
| Mortality at longest follow-up |  |  |  |  |  |
| Continuous | per SD | NA | 1.10 (1.09-1.12) | 1.07 (1.04-1.09) | <0.001 |
| Quartile | Normal | 171/731(23.4%) | 1 [Reference] | 1 [Reference] |  |
|  | Severe anemia | 34/41(82.9%) | 13.88(6.05-31.84) | 17.50(6.92-44.27) | <0.001 |
|  | Moderate anemia | 36/69(52.2%) | 3.12(1.89- 5.14) | 3.13(1.69- 5.82) |  |
|  | Mild anemia | 89/250(35.6%) | 1.58(1.16- 2.15) | 1.42(0.96- 2.09) |  |
|  | High hemoglobin | 75/199(37.7%) | 1.73(1.24- 2.41) | 1.57(1.05- 2.36) |  |

**
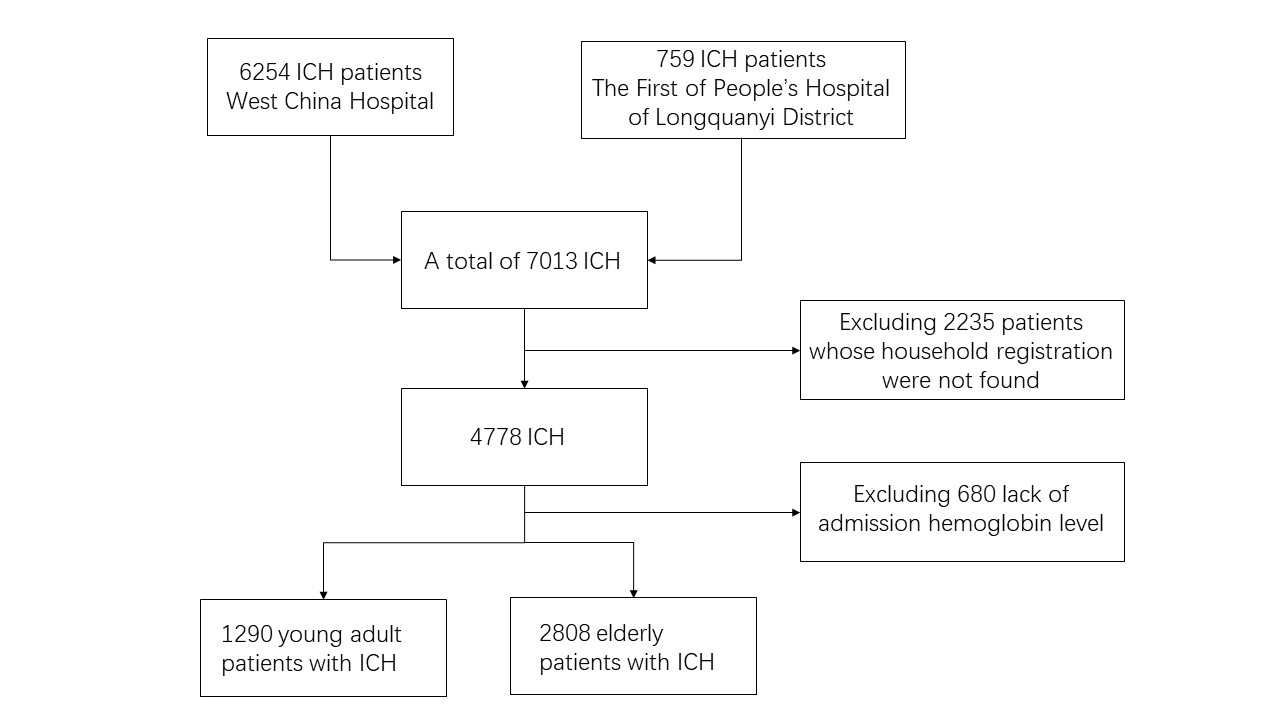
**

**eFigure 1 Flow diagram of patients included in the cohort**

**
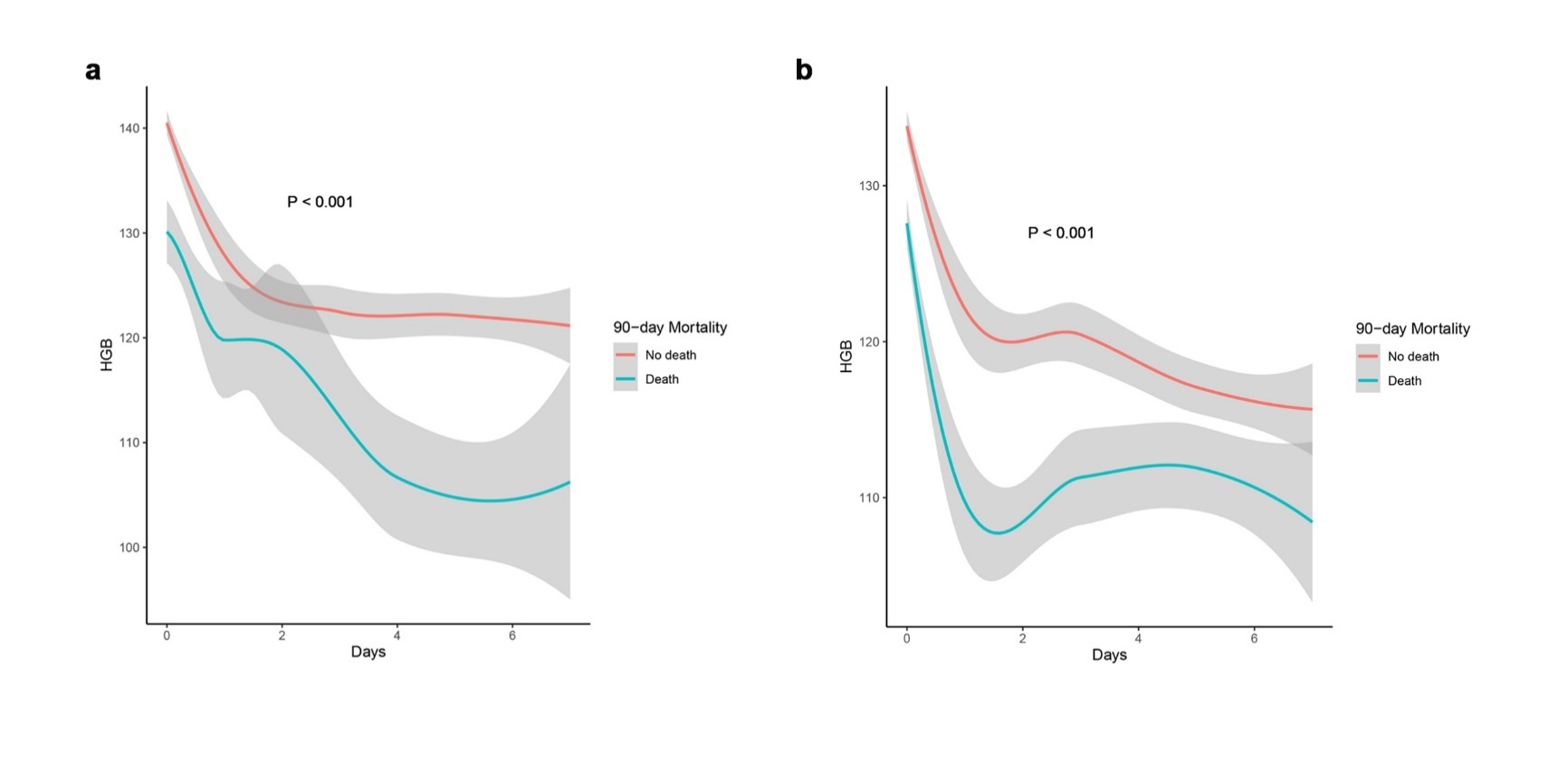
****eFigure 2 Trajectory of hemoglobin concentration within the first week after admission among young adult and elderly patients with ICH. a) young adult patients, b) elderly patients.**
